# Construction and validation of a prognostic model for tongue cancer based on three genes signature

**DOI:** 10.1097/MD.0000000000036097

**Published:** 2023-11-17

**Authors:** Haosheng Tan, Hui Huang, Huaiyu Yang, Jiaxin Qian, Liyuan Wei, Wensheng Liu

**Affiliations:** a Department of Head and Neck Surgical Oncology, National Cancer Center/National Clinical Research Center for Cancer/Cancer Hospital, Chinese Academy of Medical Sciences and Peking Union Medical College, Beijing, China.

**Keywords:** gene signature, independent prognostic factor, prognostic model, TSCC

## Abstract

Tongue squamous cell carcinoma (TSCC) has a poor prognosis and destructive characteristics. Reliable biomarkers are urgently required to predict disease outcomes and to guide TSCC treatment. This study aimed to develop a multigene signature and prognostic nomogram that can accurately predict the prognosis of patients with TSCC. We screened differentially expressed genes associated with TSCC using The Cancer Genome Atlas dataset. Based on this, we developed a new multi-mRNA gene signature using univariate Cox regression, Least Absolute Shrinkage and Selection Operator regression, and multivariate Cox regression. We used the concordance index to evaluate the accuracy of this new multigene model. Moreover, we performed receiver operating characteristic and Kaplan–Meier survival analyses to assess the predictive ability of the new multigene model. In addition, we created a prognostic nomogram incorporating clinical and pathological characteristics, with the aim of enhancing the adaptability of this model in practical clinical settings. We successfully developed a new prognostic model based on the expression levels of these 3 mRNAs that can be used to predict the prognosis of patients with TSCC. This prediction model includes 3 genes: KRT33B, CDKN2A, and CA9. In the validation set, the concordance index of this model was 0.851, and the area under the curve was 0.778 and 0.821 in the training and validation sets, respectively. Kaplan–Meier survival analysis showed that regardless of whether it was in the training or validation set, the prognosis of high-risk patients was significantly worse than that of low-risk patients (*P* < .001). Multivariate Cox regression analysis revealed that this model was an independent prognostic factor for patients with TSCC (*P* < .001). Our study suggests that this 3-gene signature model has a high level of accuracy and predictive ability, is closely related to the overall survival rate of patients with TSCC, and can independently predict the prognosis of TSCC patients with high accuracy and predictive ability.

## 1. Introduction

Tongue squamous cell carcinoma (TSCC) is the most common type of cancer in the oral cavity, accounting for over 90% of all malignant tumors in the oral cavity.^[1,2]^ The primary treatment options for tongue cancer include surgery, conventional radiotherapy, and chemotherapy. Despite advances in the treatment of TSCC, the overall 5-year survival rate remains low.^[3]^ Therefore, there is an urgent need to develop new prognostic models to identify high-risk patients with TSCC and to provide personalized treatment plans. In addition, an accurate and quantitative prognostic model is necessary to assist patients with consultation.

Increasing evidence suggests that the abnormal expression of certain messenger RNAs (mRNAs) is closely related to the prognosis of patients with TSCC, and these mRNAs can be used as molecular biomarkers for the prognostic assessment and identification of potential high-risk patients. Recently, an increasing number of molecular biomarkers have been integrated into models for the prognostic assessment of cancer patients.^[4–8]^ However, the prognostic features and nomographs of multiple mRNA in TSCC patients have not been sufficiently explored.

With the development of second-generation sequencing technology, attention has gradually turned to the analysis and alignment of high-throughput sequencing data, leading to the establishment of The Cancer Genome Atlas (TCGA) database, which provides an avenue for tumor-related research through the mining and analysis of genomic data on cancer genes. Initiated and completed by the National Cancer Institute and the National Human Genome Research Institute in the United States, TCGA is a genomic variation map of human tumors obtained through large-scale sequencing of the human tumor genome, containing information on genomics, transcriptomics, epigenetics, proteomics, as well as clinical information. It has been shown that there are significant differences in cancer-related gene expression between TSCC samples and normal tongue tissue samples.^[9]^ Therefore, we employed various methods, including univariate Cox regression, Least Absolute Shrinkage and Selection Operator (LASSO) regression, and multivariate Cox regression, to establish a prognostic model based on multiple mRNAs that could reliably and accurately predict the prognosis of TSCC patients. The accuracy of the model was evaluated by testing its performance and predicting the patient prognosis.

## 2. Methods

### 2.1. Data download and extraction

We downloaded normal and control samples for oral tongue cancer and the base of tongue cancer from the official website of TCGA at https://portal.gdc.cancer.gov/. To select the appropriate cases, we choose either “other and unspecified parts of the tongue” or “base of tongue” from the “Primary Site” menu. We selected “TCGA” as the program and “TCGA-HNSC” as the project. To minimize the impact of race on the experimental conclusions as much as possible, we only extracted information from the Caucasian population. A total of 152 samples, consisting of 137 tumor samples and 15 normal control samples, were downloaded. Each sample was analyzed for the expression of 59,427 transcripts, including both encoded and non-encoded transcripts.

### 2.2. Model construction

We used the Perl program to extract the necessary raw data and utilized the R language “limma” package for differential analysis. The extracted data type is transcripts per million (tpm), which represents the number of transcripts per million mRNA molecules in a sample. The criteria for differential gene screening were an absolute |logFC| should be >2 and FDR should be <0.01. Remove genes with an average expression value below 0.5 tpm. These differentially expressed genes (DEGs) could be considered as candidate genes for further regression analysis and prognosis model establishment. We used the “pacman” and “limma” packages in R to merge the DEGs and clinical data. The “caret” package in R was used to randomly divide the TCGA data into training and validation sets at a 1:1 ratio. In the training set, the “survival” package in R was used for univariate Cox regression analysis to obtain DEGs related to overall survival (OS), with a filtering condition of *P* < .1. The “glmnet” and “survival” packages in R were used to screen the prognosis genes by LASSO regression analysis and visualize the data to avoid overfitting. Subsequently, the “survival” and “survminer” packages in R were used for backward multivariate Cox hazard regression analysis and data visualization to select the optimal model that could predict the OS of TSCC. Finally, we calculated the mRNA-based prognostic risk score by combining the expression values of prognostic genes and their regression coefficients in the multivariate Cox regression model (backward). The formula for the risk score was as follows:


$$\begin{array}{l} Risk\;score=\sum_{i=1}^{n}Exp_{i}\times B_{i} \end{array}$$

In this study, “*i*” represents the gene in the model, “*n*” represents the number of genes in the model, “Exp_*i*_” represents the expression value of gene “*i*,” and “*B*_*i*_” represents the regression coefficient of gene “*i*” in multivariate Cox regression analysis. The training and validation sets of patients with TSCC were divided into high- and low-risk groups, based on the median risk score.

### 2.3. Verification of model accuracy

The concordance index (c-index) for the model in the validation set was calculated using the “survival” package in R, incorporating both the risk score and clinicopathological features.

### 2.4. Assessment of model prognostic ability

Time-dependent receiver operating characteristic (ROC) analysis of the training and validation sets was performed using the Kaplan–Meier “survivalROC” package in R. ROC curves were plotted, and the area under the curve (AUC) was calculated. Kaplan–Meier survival analysis and curves were plotted in R using the plan “Meier survival” package for both patient sets. Heat maps for risk stratification were generated using the “pheatmap” package in R. The distribution of risk scores and corresponding survival curves were plotted in R using the base package.

### 2.5. Clinical application of the model

Based on prognostic gene model and clinical pathological features, a multivariate Cox proportional hazards regression analysis (backward) and data visualization were performed using R packages “survival” and “survminer” to select features for constructing a forest plot. Subsequently, a forest plot predicting OS of patients with TSCC was constructed based on the selected features.

### 2.6. Statistical analysis

Unless otherwise specified, all significance tests were 2-tailed, and a *P* value <0.05 was considered statistically significant.

## 3. Results

### 3.1. Clinical characteristics of TSCC patients in TCGA

We collected data on 137 patients from TCGA database, including 94 males and 43 females. The median age was 60 years (range 19–87), with 39 patients aged 65 years or older. According to the AJCC staging system, there were 12, 24, 32, and 69 cases of stages I and II disease, 32 cases of stage III disease, and 69 cases of stage IV disease, respectively. The primary tumor sites were the oral tongue in 108 patients, the base of the tongue in 25 patients, and the floor of the mouth in 4 patients. Lymph node involvement was observed in 69 patients. No distant metastases were detected in any patient.

### 3.2. Identifying genes with differential expression compared to normal tissue

DEGs were identified and compared to normal tissue using the “limma” package under the conditions specified in Section 2. After filtering out genes with average expression below 0.5 tpm, a total of 1009 genes remained and were selected for further regression analysis. Integration of RNA expression data and clinicopathological features was conducted using the “pacman” and “limma” packages in the R language. The cases were randomly divided into training and testing sets at a ratio of 1:1, according to their clinicopathological characteristics (Table 1).

**Table 1:** Clinical and pathological characteristics of patients included in the training and validation sets.

	Training set (n = 69)	Validation set (n = 68)
Gender		
Male	45 (65.2%)	49 (72.1%)
Female	24 (34.8%)	19 (27.9%)
Age	61 (24–87)	59 (19–79)
TNM stage		
I	5 (7.2%)	7 (10.3%)
II	9 (13.0%)	15 (22.1%)
III	16 (23.2%)	16 (23.5%)
IV	39 (56.5%)	30 (44.1%)
T		
T1	9 (13.0%)	12 (17.6%)
T2	17 (24.6%)	29 (42.6%)
T3	26 (37.7%)	17 (25.0%)
T4	17 (24.6%)	10 (14.7%)
N		
N−	25 (36.2%)	31 (45.6%)
N+	44 (63.8%)	37 (54.4%)
M		
M0	67 (97.1%)	66 (97.1%)
Unknown	2 (2.9%)	2 (2.9%)
Grade		
1	9 (13.0%)	7 (10.3%)
2	43 (62.3%)	42 (61.8%)
3	13 (18.8%)	14 (20.6%)
4	3 (4.3%)	1 (1.5%)
Unknown	1 (1.5%)	4 (5.9%)

### 3.3. Univariate Cox regression analysis of DEGs

We used the Kaplan–Meier “survival” package in R to conduct univariate Cox regression analysis on the training set to evaluate the role of candidate DEGs in TSCC prognosis. Among the 1009 genes in the training set, 46 were associated with the prognosis (Table 2).

**Table 2:** The 46 significantly differentially expressed cancer-related genes (*P* < .1) identified by univariate Cox regression analysis.

Gene	HR	HR.95L	HR.95H	*P* value
CA9	1.024016358	1.012496746	1.035667033	3.93E−05
TRIML2	1.300973504	1.103879757	1.533257629	.001694864
ITGA5	1.022049739	1.00677994	1.037551134	.004514808
GDPD2	1.0735098	1.017102969	1.13304486	.010001941
LAMA3	1.005476158	1.001305211	1.00966448	.010024836
TH	1.422568449	1.078327701	1.876703149	.012650843
AL161431.1	1.032498072	1.006532799	1.059133165	.013853203
AL354953.1	1.198633429	1.035982466	1.386820862	.014889035
ODAPH	1.220866665	1.03473383	1.440481958	.018053127
CYP27B1	1.134017646	1.021392854	1.25906111	.018443535
CNGB1	1.08216928	1.013287873	1.155733115	.01860487
GAST	1.017952366	1.002965692	1.033162977	.018708548
LHX5	2.113999673	1.115794665	4.005212391	.021674532
MMP9	1.004716251	1.000637522	1.008811605	.023388006
KRT33B	0.001903586	8.33E-06	0.435117625	.023807503
PAEP	1.145259852	1.015352815	1.291787553	.027244322
CALB1	1.049664694	1.005452671	1.095820819	.027269725
LAMC2	1.001280211	1.00014011	1.002421612	.027737159
CDKN2A	0.947829554	0.903374665	0.994472061	.028805774
AP003555.1	1.215374755	1.016702801	1.452868817	.032204611
CXCL13	0.967090447	0.937241819	0.997889673	.036435338
AC022031.2	1.791989408	1.035579931	3.10089636	.037076236
ARTN	1.116988563	1.006501703	1.239603914	.037350259
ADGRE1	1.324223844	1.012973754	1.731109797	.039950809
FUT6	0.68343512	0.472803881	0.987901288	.042896616
SERPINH1	1.010782431	1.000313135	1.021361298	.043497437
NELL2	1.018427658	1.000357473	1.036824258	.045598289
AC007991.2	0.404799759	0.166448729	0.984464377	.04609794
SH2D5	1.114634716	0.99985049	1.24259633	.05031604
INHBA	1.014426957	0.999450908	1.029627412	.05908164
PDPN	1.00867871	0.999362056	1.01808222	.067974344
WNT7A	1.087512863	0.993340702	1.190612873	.069465464
PCDHGC5	1.158811559	0.988241974	1.358821285	.069620783
FKBP9P1	1.273469995	0.980667923	1.653695191	.069751807
GPR39	1.245385473	0.977686585	1.586382591	.075533884
FOLR3	1.037944428	0.995708576	1.081971834	.078907232
IL36A	0.953314247	0.903469014	1.005909488	.080998263
SCG5	1.142537779	0.98307906	1.327861236	.082308297
LOXL2	1.021457128	0.996942448	1.04657462	.086731654
DYNAP	0.583507275	0.313380429	1.086477359	.089420874
SPRR3	0.998648956	0.997073538	1.000226863	.093276548
GRIN2D	1.104196903	0.983255605	1.240014087	.094000533
FSTL3	1.011158439	0.998040479	1.024448818	.095801162
MATN3	1.240924347	0.962235225	1.600329309	.09624776
TMPRSS11B	0.823560965	0.653976689	1.037120549	.098916313
SFTA2	0.000490182	5.68E-08	4.233225772	.099366504

### 3.4. A prognostic model containing 3 genes was established using the LASSO regression and stepwise multivariate Cox hazard ratio regression methods

**Figure 1.**
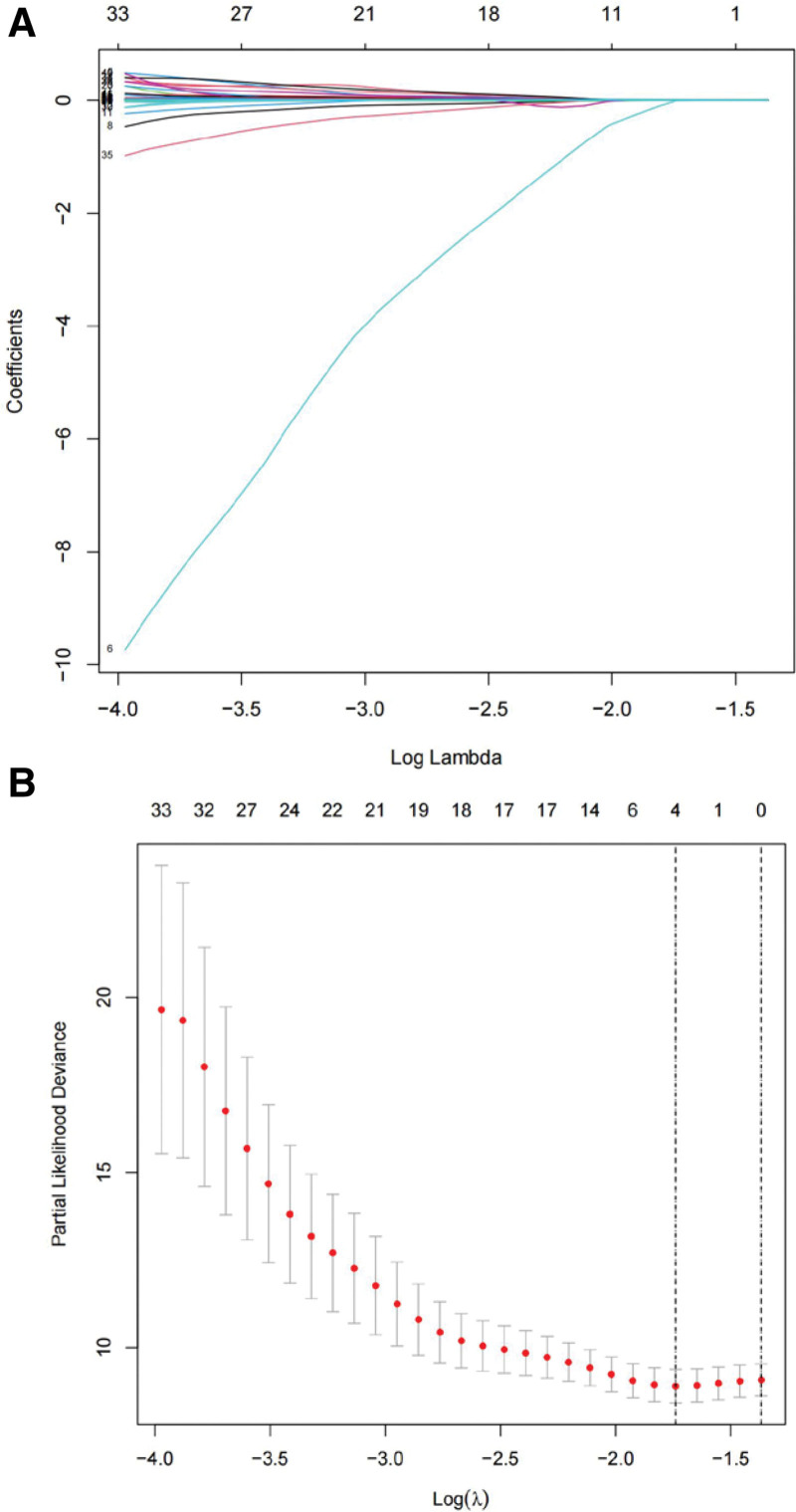
LASSO regression. (A) Regression analysis using LASSO-penalized logistic regression with parameter *λ* adjusted by 10-fold cross-validation. (B) The sum of coefficients *β*_*i*_ obtained by LASSO feature selection represents the coefficient sum of the LASSO model. LASSO = Least Absolute Shrinkage and Selection Operator.

**Figure 2.**
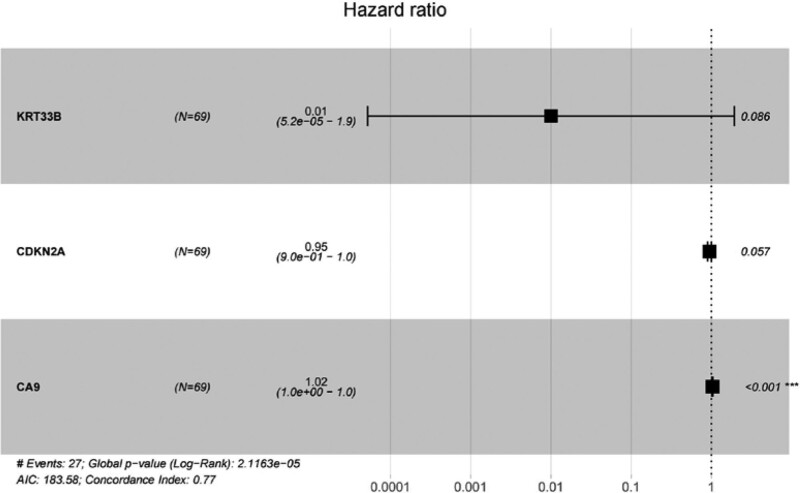
Forest plot of the prognostic model constructed by stepwise multivariate Cox regression analysis, including 3 genes.

Incorporating the 46 genes selected by univariate Cox regression analysis, LASSO regression analysis was applied by regularization with a penalty term to avoid overfitting and select the most important features. The regularization parameter lambda was set to log lambda. min. Four genes, KRT33B, cyclin dependent kinase inhibitor 2A (CDKN2A), carbonic anhydrase 9 (CA9), and ITGA5, were ultimately chosen as statistically significant (Fig. 1) and incorporated into the multivariate Cox regression model. Subsequently, a prognostic model including 3 genes, KRT33B, CDKN2A, and CA9, was established (Fig. 2) based on their expression levels and their association with prognosis in TSCC, which were depicted in the Figure S1, Supplemental Digital Content, http://links.lww.com/MD/K753 and Figure S2, Supplemental Digital Content, http://links.lww.com/MD/K754.

Risk Score = −4.605689081 × KRT33B expression − 0.054674985 × CDKN2A expression + 0.022976245 × CA9 expression

### 3.5. Validation of model accuracy and predictive ability

**Figure 3.**
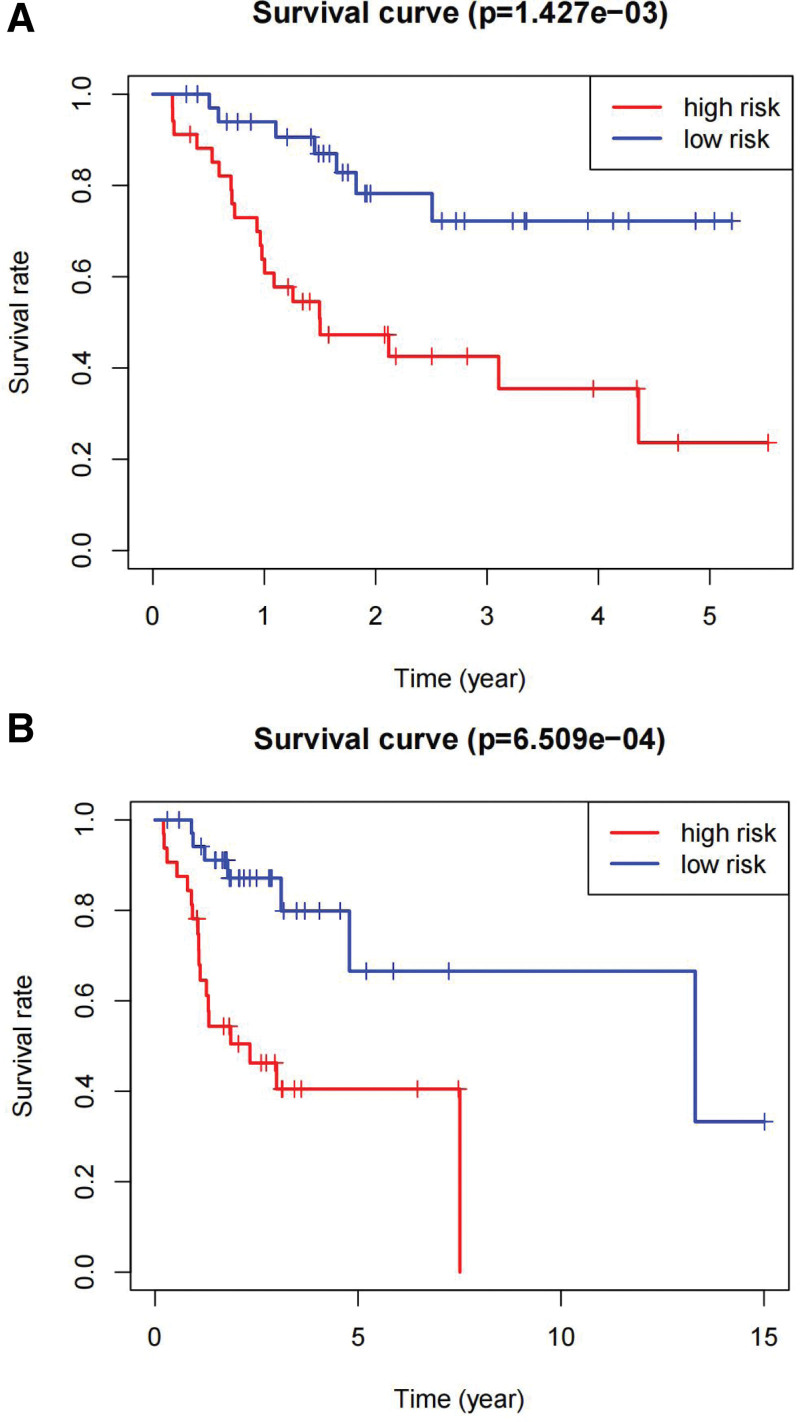
Kaplan–Meier survival curve based on risk score model of the training set: (A) training set and (B) testing set.

**Figure 4.**
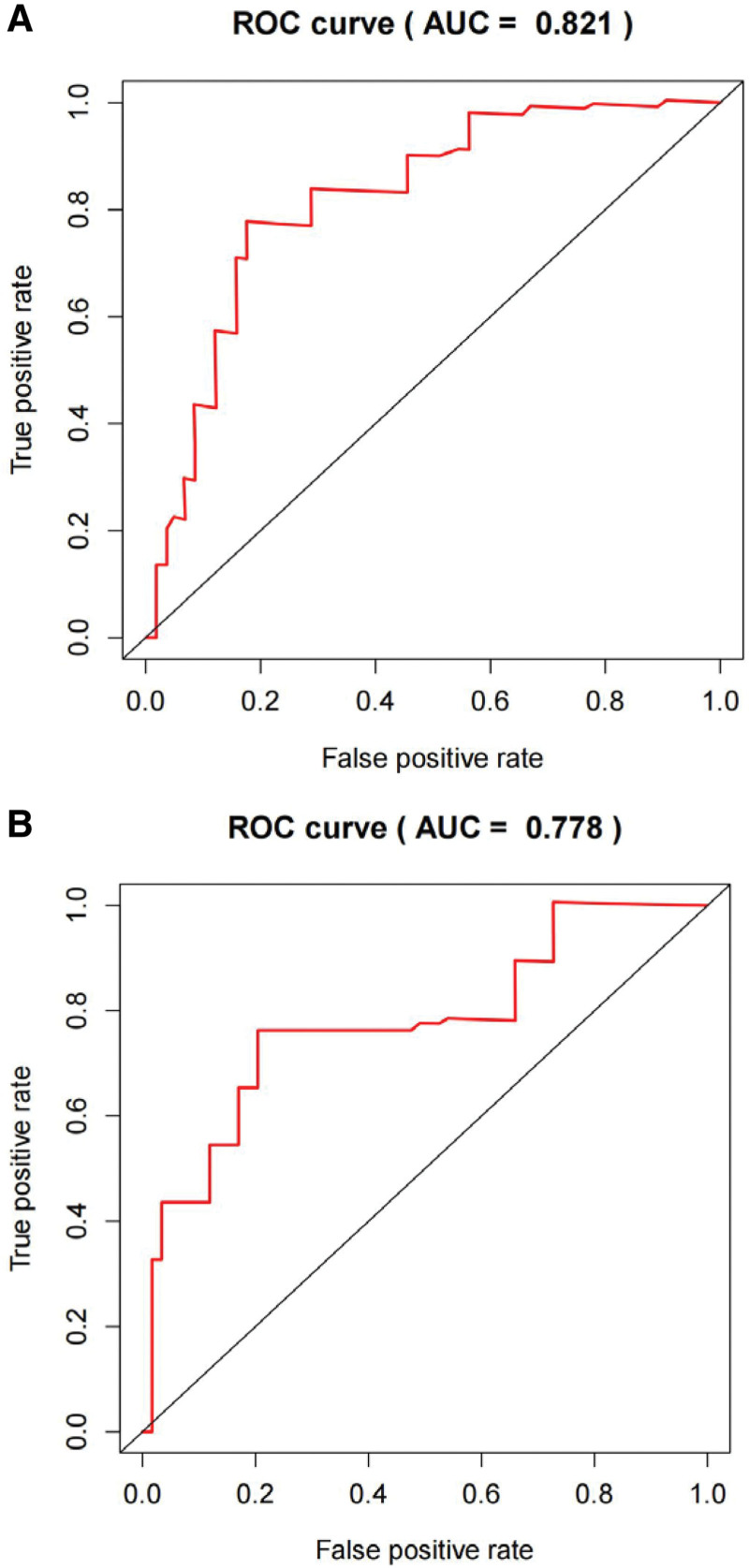
ROC analysis based on risk score model of the training set: (A) training set and (B) testing set. ROC = receiver operating characteristic.

**Figure 5.**
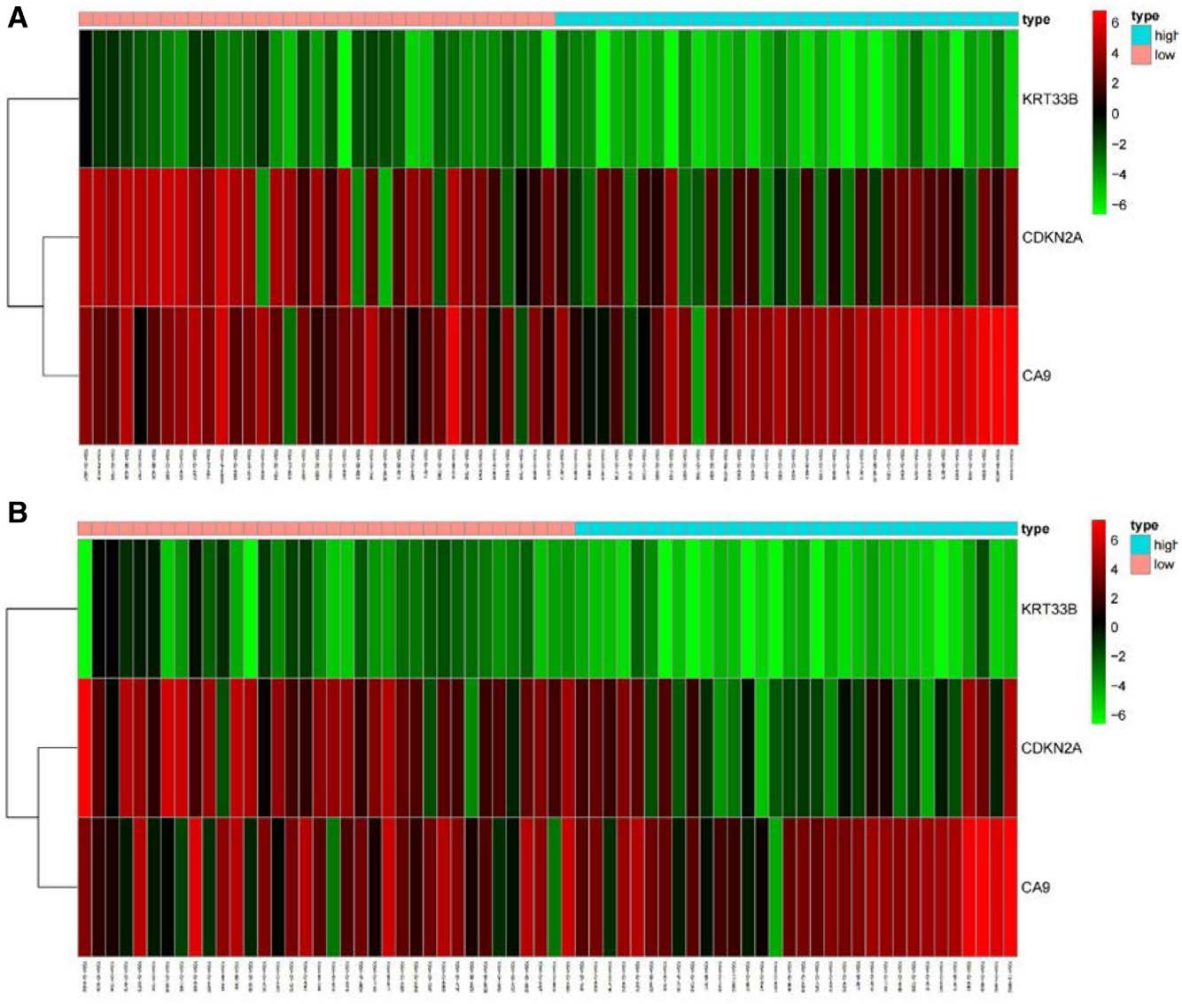
Heatmap based on risk score model of the training set: (A) training set and (B) testing set. Red represented high expression and green represented low expression. The number next to the color bar represented the value of log2(TPM + 0.01).

**Figure 6.**
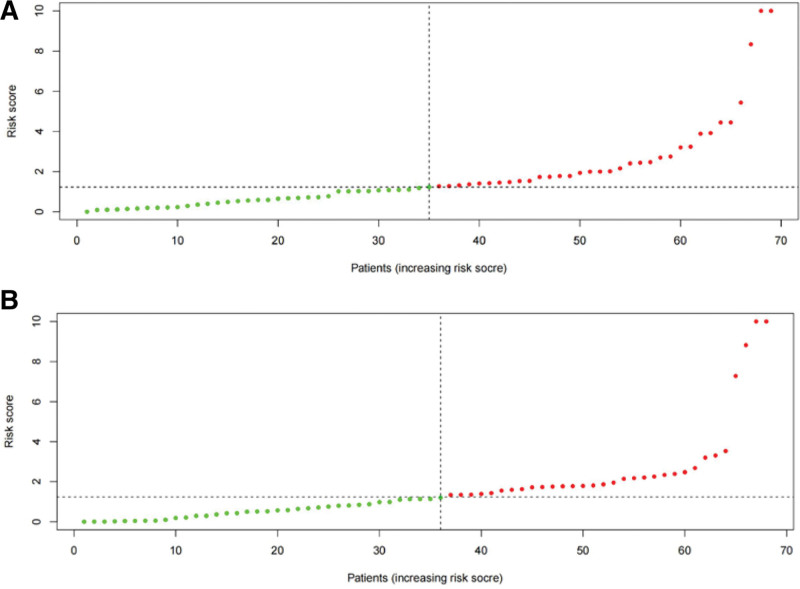
Risk score distribution based on risk score model of the training set: (A) training set and (B) testing set.

**Figure 7.**
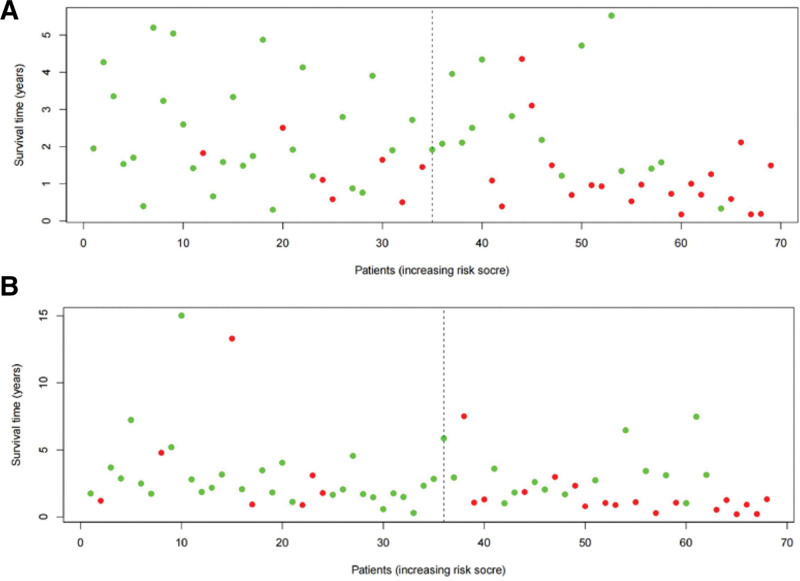
Risk score survival based on risk score model of the training set: (A) training set and (B) testing set.

The risk score of each patient in the training and validation sets was calculated according to the risk score formula, and patients in each set were divided into high- and low-risk groups based on the median risk score. The c-index of the validation set was 0.851, indicating high accuracy of the model. KM survival analysis showed that patients with high-risk scores had a significantly worse prognosis than those with low-risk scores in both the training and validation sets (Fig. 3A and B, *P* < .001). The 5-year survival rate for high-risk individuals in the training set was 23.6%, whereas it was 72.2% for the low-risk group (*P* = .001). In the validation set, the 5-year survival rate for high-risk individuals was 40.5%, compared to 66.6% for the low-risk group (*P* = .001). The ROC analysis showed that the AUC in the training and validation sets were 0.821 and 0.778, respectively (Fig. 4A and B). This indicates that the model has a high predictive ability and accuracy. Through the risk heatmap of the training and validation sets (Fig. 5), we found significant differences in the expression levels of the 3 genes between the high-risk and low-risk groups. The risk score distribution chart (Fig. 6) shows that the risk score was linearly distributed. The risk score survival chart (Fig. 7) showed that the mortality risk of the high scorers significantly increased. These results suggest that our predictive model has a high predictive ability and stability.

### 3.6. Clinical application of the model

**Figure 8.**
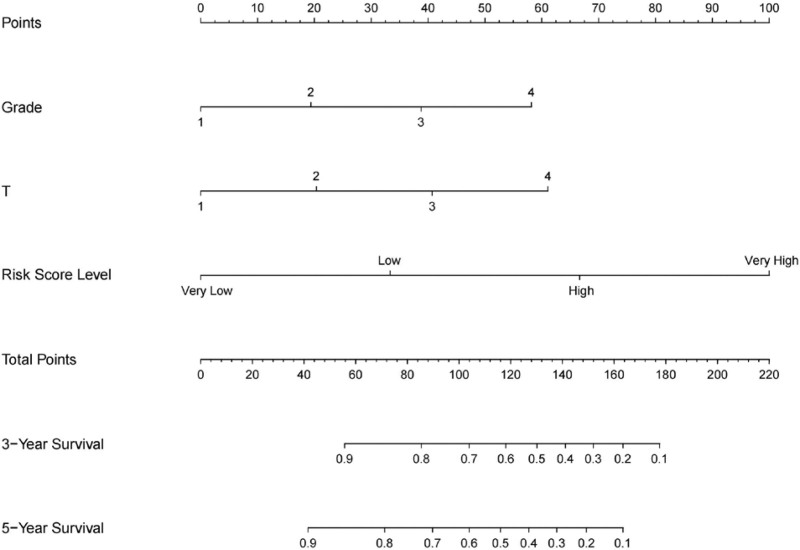
Column chart predicting the overall 3-year and 5-year survival of TSCC patients by integrating risk groups and clinicopathological features. TSCC, tongue squamous cell carcinoma.

Patients were divided into 4 risk groups according to quartiles of the risk score: extremely low-risk, low-risk, high-risk, and extremely high-risk. Based on the risk group and clinicopathological features, a multivariate Cox regression analysis (backward) was performed on TSCC patients, and 3 risk factors for TSCC were ultimately identified: grade, T, and risk score (Table 3). All 3 risk factors had 4 grades each (Grade 1–4, T 1–4, 4 risk score levels). Compared to Grade and T, our model has an HR of 2.325, which is significantly higher than the other 2 risk factors, indicating that our model has a greater advantage in predicting prognosis compared to Grade and T. A survival curve was constructed to predict the OS of patients with TSCC using these 3 risk factors (Fig. 8), thus providing a valuable tool for clinical decision-making.

**Table 3:** Three characteristics selected for constructing the survival curve by multivariable Cox regression analysis.

Characteristic	Coef	HR	HR.95L	HR.95H	*P* value
Grade	0.490863226	1.633725886	1.043562407	2.557643177	.031839576
T	0.515404107	1.674314968	1.211713554	2.313525835	.001784667
Risk score level	0.843801703	2.325189877	1.715320317	3.151894087	5.43E−08

## 4. Discussion and conclusion

We established a prognostic model based on only 3 genes to predict the prognosis of patients with TSCC. The predictive power of this model was stronger than that of the other clinicopathological factors, with an HR of 2.325 (95% confidence interval: 1.715–3.152). To evaluate the accuracy and reliability of this model, we conducted c-index tests, ROC analyses, and KM analyses, all of which showed that the model had good predictive accuracy. Therefore, we believe that this 3-gene model can effectively predict the prognosis of TSCC patients. Furthermore, to make this model more intuitive and practical, we created a column chart integrating risk assessment, grading, and T staging to predict the prognosis.

Our model includes 3 genes: KRT33B, CDKN2A, and CA9. KRT33B, also known as keratin 33 B, is a member of the keratin family. In normal tissues, keratin encoded by this gene promotes the growth and maintenance of hair and nails. Keratins are a large class of proteins produced by keratinocytes in the skin, hair, nails, and other cornified cells, and are closely related to the occurrence and development of SCC.^[10–13]^ Studies have shown that KRT33B is significantly suppressed in low-grade gliomas and is associated with poor prognosis,^[14]^ suggesting that this protein may be a marker for differentiation status, with high expression indicating better differentiation and prognosis. This is consistent with our results (Figure S1A, Supplemental Digital Content, http://links.lww.com/MD/K753 and Figure S2A, Supplemental Digital Content, http://links.lww.com/MD/K754), where patients with low KRT33B expression had significantly worse outcomes than those with high expression. CDKN2A, also known as cyclin dependent kinase inhibitor 2A/p16, plays an important role in the occurrence and development of head and neck squamous cell carcinoma (HNSCC) and is a well-known tumor suppressor gene, particularly in HPV-related HNSCC.^[15,16]^ Our study also found that low CDKN2A expression in TSCC was significantly associated with a poor prognosis (Figure S1B, Supplemental Digital Content, http://links.lww.com/MD/K753 and Figure S2B, Supplemental Digital Content, http://links.lww.com/MD/K754), indicating the importance of CDKN2A in TSCC. CAs are a class of large zinc metalloenzymes that catalyze the reversible hydration of carbon dioxide and participate in a variety of biological processes. CA9, also known as carbonic anhydrase 9, is one of the only tumor-associated isozymes in the CA family.^[17]^ A study on gliomas found that targeting this gene can effectively inhibit tumor growth.^[18]^ Wang et al found that high CA9 expression in TSCC is closely related to poor pathological T staging and warrants further exploration as a potential prognostic biomarker and therapeutic target for OTSCC.^[19]^ Guan et al^[9]^ also found that CA9 plays an important role in TSCC prognosis and tumor grading and that its expression is closely related to the regulation of cell differentiation, multiple oncogenes, and pathways related to cancer. Our research found that CA9 is also related to the prognosis of TSCC patients, with poorer prognosis in high expressers (Figure S1C, Supplemental Digital Content, http://links.lww.com/MD/K753 and Figure S2C, Supplemental Digital Content, http://links.lww.com/MD/K754). All 3 genes mentioned above are potentially closely related to the occurrence and development of TSCC, and further in-depth research may provide a theoretical basis and significant breakthroughs for treatment and prognosis monitoring.

Gene expression prediction models have been used to predict the probability of related diseases or other phenotypes by analyzing the gene expression. Such models have demonstrated a good predictive performance and can provide personalized medical services and treatment plans for different patients, thereby contributing to improved treatment efficacy and lower costs. However, gene expression prediction models also have limitations, mainly evident in their tendency to overfit, resulting in poor predictive performance on testing datasets. In addition, gene expression-based prediction models tend to produce many gene variables that lack intuitive and easy-to-understand indicators, making it challenging to transform these variables into clinical indicators. Nonetheless, in recent years, researchers have established an increasing number of multiple biological markers and prognostic nomograms and applied them to clinical decisions and prognostic evaluations of different cancer types.^[20–22]^ However, studies of gene expression prediction models for TSCC are limited. Montero et al^[23]^ constructed a prognostic nomogram for patients with oral cancer, which included some important clinicopathological variables, such as age, sex, race, alcohol and smoking history, subsite of the oral lesion, invasion of other structures, tumor size, and clinical lymph node status. However, biological markers were not included in the model. The c-index of the model was only 0.67. Subsequently, Bobdey et al^[24]^ developed a prognostic model for patients with oral cancer with a c-index of 0.7263; however, biological markers were not included. Jiang et al^[25]^ integrated GEO and TCGA datasets to establish an OTSCC prognostic model that included 16 genes; however, the c-index of this model for predicting OS was only 0.652, suggesting a relatively low predictive ability. Recently, Liu et al established a prognostic model consisting of 15 genes with an internal validation c-index of 0.849 and an external validation c-index of 0.804. However, this model was only targeted for oral tongue cancer and did not include floor of the mouth and tongue root cancers, limiting its scope of application.^[26]^

The above model either predicts a low performance or contains too many genes. Currently, no prediction model includes a gene signature/biomarker for all patients with TSCC. Therefore, the model we established can predict the prognosis of all tongue cancers, including tongue root and base cancer. The model contained only 3 genes closely related to cancer development, but the c-index (0.851) and AUC (0.821) reached high levels, which can accurately predict the progression of the disease and greatly improve the accuracy of clinical diagnosis. This method is expected to become a part of precision medicine. Second, our model contained only 3 genes with high operability, which greatly reduced the complexity of clinical testing and improved the stability and accuracy of the results, thereby improving the efficiency of clinical diagnosis. In the future, it will likely become an important tool for predicting TSCC prognosis of TSCC. To reduce genetic collinearity and improve the accuracy of the results, this study used LASSO stepwise regression analysis before conducting a multiple factor Cox regression to improve the predictive accuracy of the model. Finally, we established a prognostic nomogram that integrates this predictive model with clinicopathological features, making the results more intuitive, easy to understand, and easy to operate, which will help promote the application of this model in clinical practice. Our model, as the most advantageous prognostic factor, can predict the prognosis of patients with TSCC more accurately when combined with clinical and pathological data. However, owing to the lack of data on patients with distant metastasis in TCGA, the M stage/TNM stage were assigned a lower weight in the nomogram. Only T stage was included as a prognostic factor. It is widely known that TNM staging has a significant impact on the prognosis of patients, especially M status. Therefore, in future clinical validation, TNM staging/M staging should be considered when designing nomograms. Further prospective studies are required to confirm our findings.

## Author contributions

**Conceptualization:** Haosheng Tan, Huaiyu Yang, Wensheng Liu.

**Data curation:** Haosheng Tan, Hui Huang, Huaiyu Yang.

**Formal analysis:** Haosheng Tan, Huaiyu Yang, Liyuan Wei.

**Funding acquisition:** Wensheng Liu.

**Methodology:** Haosheng Tan, Wensheng Liu.

**Supervision:** Wensheng Liu.

**Validation:** Jiaxin Qian.

**Visualization:** Haosheng Tan, Huaiyu Yang.

**Writing – original draft:** Haosheng Tan.

**Writing – review & editing:** Wensheng Liu.

## Supplementary Material




