# Supplementary material for: Construction and validation of a prognostic model for tongue cancer based on three genes signature
Source: Medicine (Baltimore). 2023 Nov 17;102(46):e36097. doi: 10.1097/MD.0000000000036097 (PMC10659661; doi:10.1097/MD.0000000000036097)
Supplement: Supplementary file 2 [file medi-102-e36097-s002.docx]

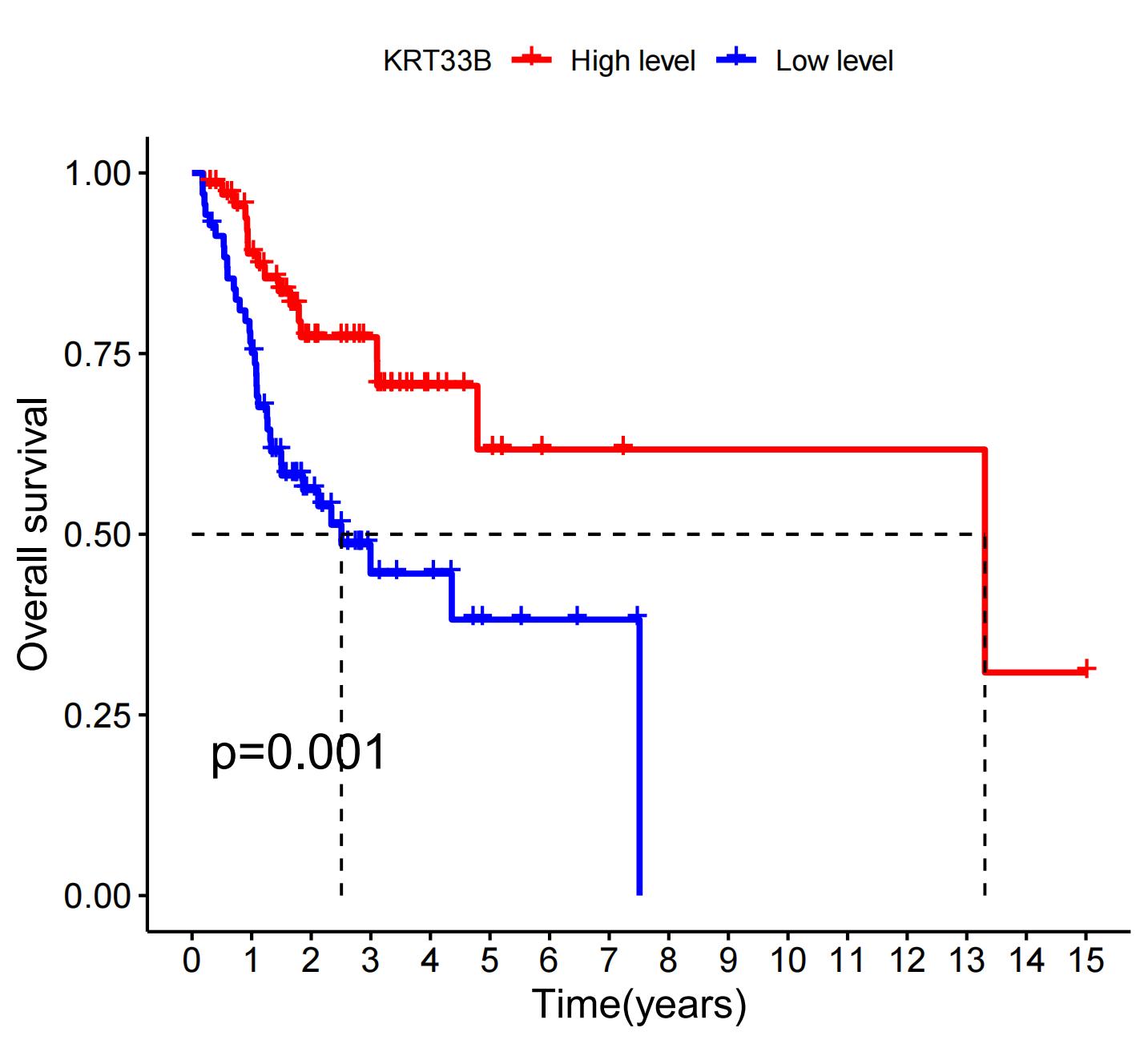


A


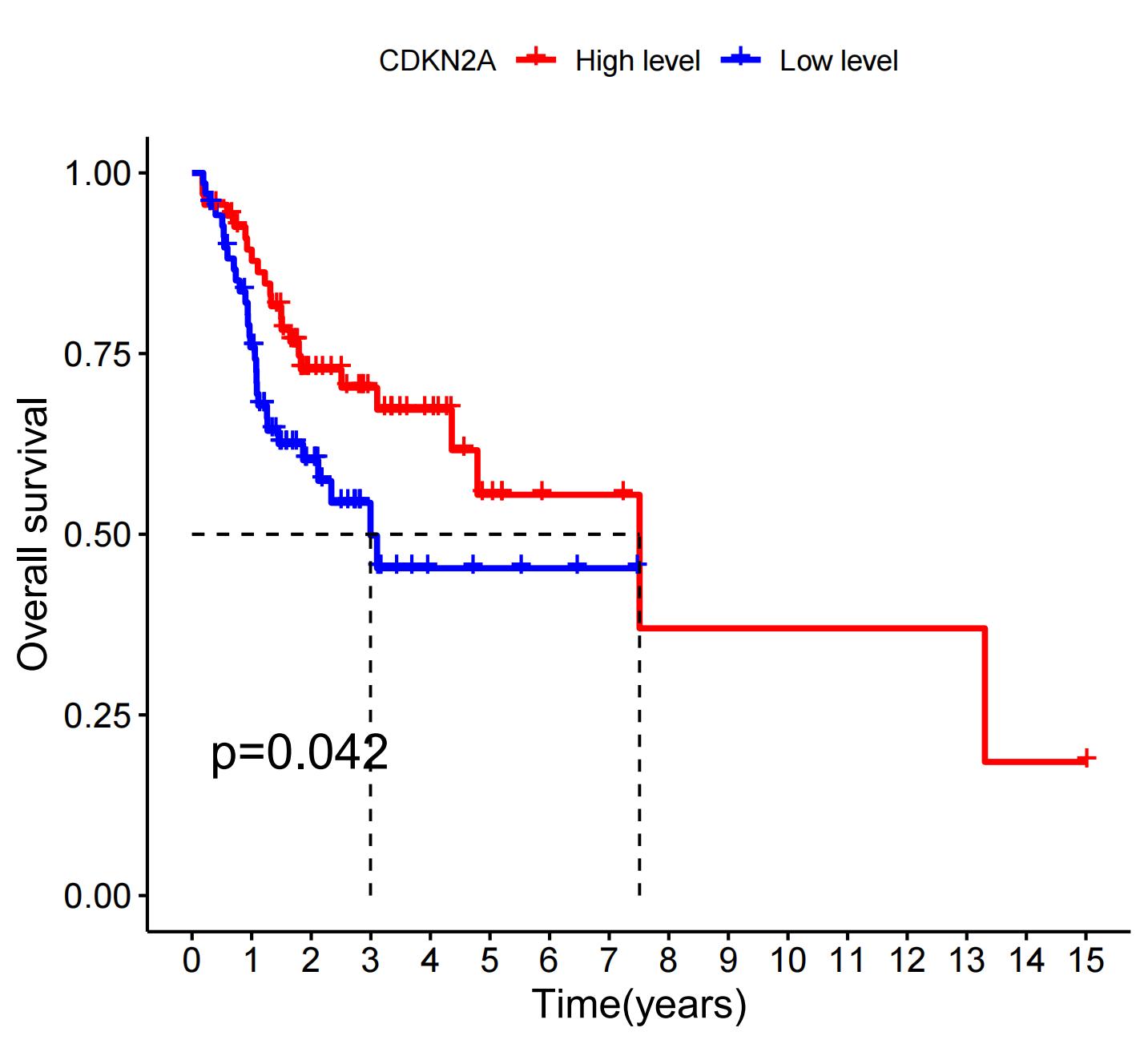


B


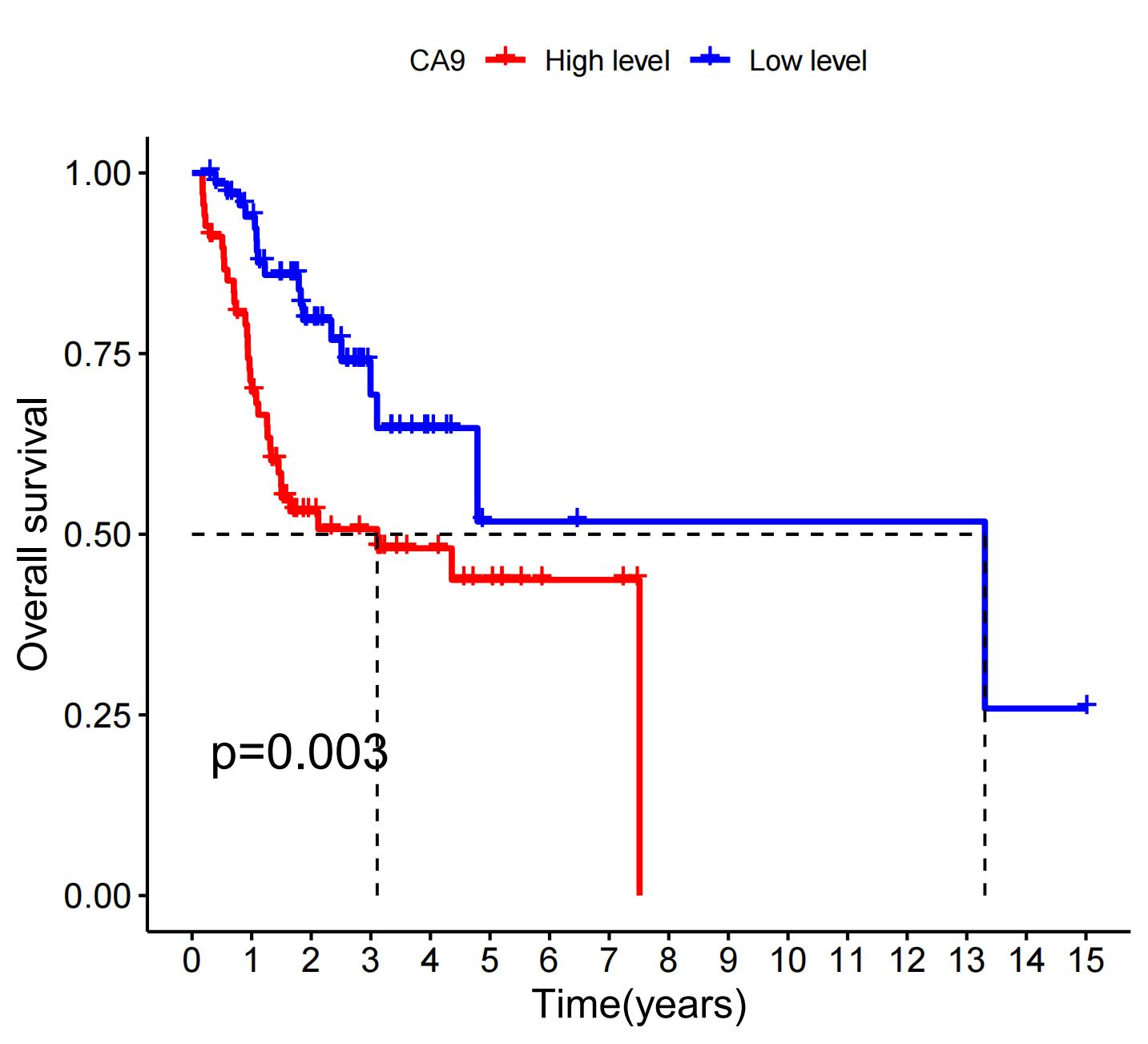


C

Supplementary Figure 2. The relationship between the expression of KRT33B, CDKN2A, and CA9 included in the model and the prognosis of TSCC patients.

1. KRT33B. (B) CDKN2A. (C) CA9.
